# Integration of epigenetics into ecotoxicology: insights and fundamental research needs

**DOI:** 10.1111/brv.70105

**Published:** 2025-11-16

**Authors:** Albano Pinto, Jana Asselman, Patrícia Pereira, Joana Luísa Pereira

**Affiliations:** ^1^ CESAM – Centre for Environmental and Marine Studies, Department of Biology University of Aveiro Campus de Santiago Aveiro 3810‐193 Portugal; ^2^ Blue Growth Research Lab Ghent University Bluebridge Building, Ostend Science Park 1 Ostend 8400 Belgium

**Keywords:** environmental epigenetics, DNA methylation, histone modifications, non‐coding RNA, epigenetic mechanisms, invertebrates, ecotoxicology

## Abstract

Epigenetics refers to heritable changes in genome function that occur without direct alterations to the DNA sequence. A multitude of environmental contaminants can influence the epigenetic marks of a genome. Changes of epigenetic marks including DNA methylation, histone modifications, and non‐coding RNAs can induce alterations at the gene transcription level, potentially leading to physiological long‐term changes that can be inherited transgenerationally. (Eco)Toxicoepigenetics is thus an emerging field of research focusing on linking environmental exposure with epigenome alterations, with a high postulated relevance for improved ecological risk assessment at the regulatory level. Despite its huge potential, fundamental knowledge is scarce and scattered concerning epigenetic regulation in relevant ecotoxicological model species and mechanisms of interaction between environmental contaminants and the epigenome. This is a paramount challenge for the efficient implementation of (eco)toxicoepigenetics that is not often recognised in the literature. Herein, we provide updated knowledge regarding the main epigenetic modifications that occur on ecotoxicologically relevant models and summarize the differences in epigenetic patterns between vertebrate and invertebrate species that are routinely used as test organisms in ecotoxicology. We also systematically revise what is known on the mechanisms through which environmental contaminants can modulate the epigenome, using three legacy contaminants of the aquatic compartment for which appreciable information exists concerning ecotoxicologically relevant species. Future directions for (eco)toxicoepigenetics research are discussed in the context of the existing knowledge, with particular emphasis on the much‐needed characterization of the epigenomes of ecotoxicological models and the need to understand better the mechanisms underlying the modulation of epigenetic marks and related machinery by environmental contaminants. This review will hopefully stimulate future research contributing to the continuous incorporation of epigenetic studies in ecotoxicology and the development and implementation of effective epigenetic‐based ecotoxicological biomarkers for environmental stress assessment.

## I. INTRODUCTION

Epigenetics refers to heritable changes in gene expression that do not directly alter DNA sequences (Bollati & Baccarelli, 2010; Cheng, Choudhuri & Muldoon‐Jacobs, 2012). The whole set of epigenetic marks that exist in a genome is often referred to as the epigenome. This array of epigenetic modifications is capable of collectively regulating the expression of genes, therefore leading to changes in the phenotype without causing changes to the genotype (Bollati & Baccarelli, 2010; Cheng *et al*., 2012; Belzil, Katzman & Petrucelli, 2016). In addition to regulating gene expression, epigenetic modifications have several other important biological functions, such as maintaining genomic stability, cell differentiation and embryo morphogenesis (Nilsson, Sadler‐Riggleman & Skinner, 2018; Hu & Yu, 2019).

The dysregulation of epigenetic mechanisms is also often associated with many diseases (Cheng *et al*., 2012; Poh, Wee & Gao, 2016). For example, altered DNA methylation patterns often lead to loss of expression of essential genes and increased genomic instability, and have been linked with various diseases such as neurodevelopmental impairment (Levy *et al*., 2022), neurodegeneration and other neurological disorders (Lu *et al*., 2013; Wen *et al*., 2016; Wolf *et al*., 2016), autoimmunity (Zouali, 2021) and various types of cancer (Pappalardo & Barra, 2021; Mensah *et al*., 2021).

Epigenetic marks are constitutively dynamic and are influenced by both intrinsic (e.g. age, sex, and genetic polymorphisms) and extrinsic (e.g. environmental exposures, stress and diet) factors (Provençal & Binder, 2015; Jeremias *et al*., 2018; Leung *et al*., 2018). Changes in the epigenome, as a response to internal and external stimuli an organism is exposed to, determine the response of cells to these stimuli. They modulate major genomic and cellular processes, which have a direct role in health outcomes not only across the lifespan of the exposed organism but also of its descendants, potentially having cascading effects on food webs and ecosystems (Cheng *et al*., 2012; Skinner *et al*., 2013; Belzil *et al*., 2016; Jeremias *et al*., 2018; Bošković & Rando, 2018). Patterns of epigenetic modifications are then inheritable, both mitotically and meiotically. These features render epigenetic marks the potential to serve as biomarkers of an individual's environment (Bowers & McCullough, 2017; Jeremias *et al*., 2018).

The role of epigenetics in ecotoxicology studies has been gaining relevance in recent years. This is not only due to the potential of epigenetic marks as reliable molecular biomarkers of exposure and effects (Jeremias *et al*., 2020), but also and particularly because epigenetic marks can provide early cues of long‐term effects. For example, transgenerational studies have linked contaminant exposures to transfer of inheritable epigenetic marks through the germline even when the external trigger that caused an epigenetic alteration has disappeared (e.g. Harney *et al*., 2022; Jeremias *et al*., 2018; Valles *et al*., 2020). In microevolution studies focusing on species adaptation strategies to less‐favourable environmental conditions through changes in their epigenetic landscape, the field also has gained significant traction (Augustyniak *et al*., 2016).

A variety of environmental factors can induce changes in the epigenome of exposed organisms, including several environmental contaminants that threaten aquatic ecosystems like cyanotoxins (Laugeray *et al*., 2018), metals (Jeremias *et al*., 2022), microplastics (Song *et al*., 2022), and pharmaceuticals (Lee *et al*., 2018). Exposure to environmental factors can lead to epigenetic (local) or epigenomic (global) effects that alter the expression of exposure‐responsive genes, which often have important roles in the development and physiology of organisms (Caldji *et al*., 2011; Feng & Lazar, 2012; Holloway *et al*., 2012; Haws, Leech & Denu, 2020).

**Fig. 1:**
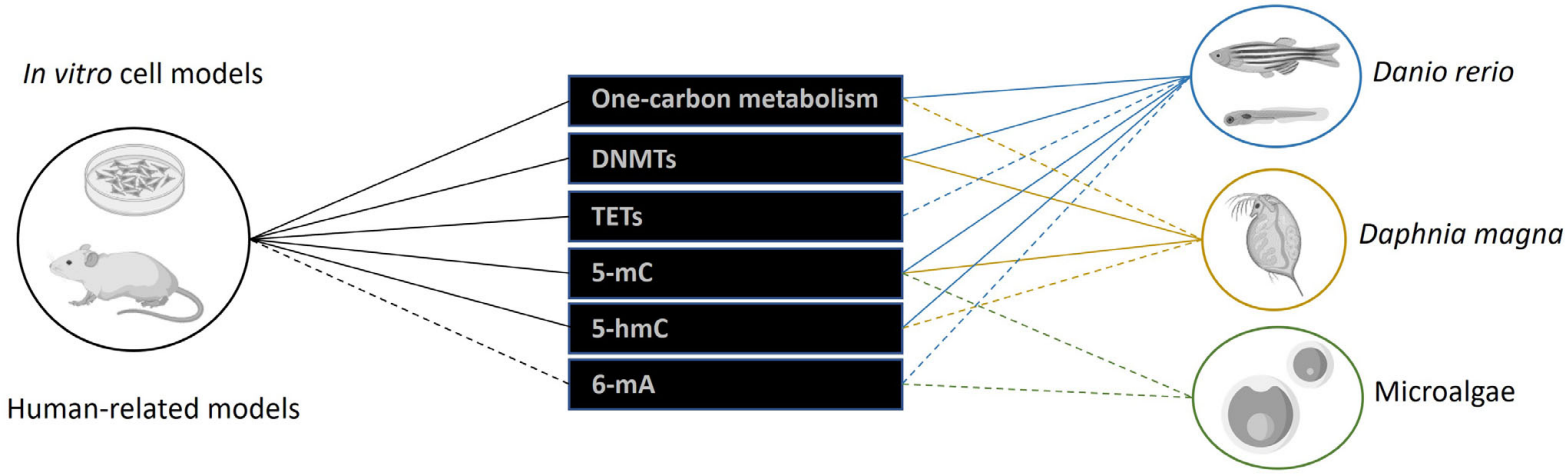
Summary of the available information in human‐related models and three common models used in ecotoxicology and regulatory Ecological Risk Assessment about epigenetic responses to environmental contaminants *via* DNA methylation (i.e. the essential pathways and enzymatic machinery involved in this form of epigenetic regulation). Epigenetic responses are divided into metabolic pathways and enzymatic machinery essential for the establishment, maintenance and removal of epigenetic marks on DNA [one‐carbon metabolism, DNA methylation ‘writer’ enzymes (DNA methyltransferases, DNMTs) and ‘eraser’ enzymes (ten‐eleven translocation methylcytosine dioxygenase, TET)] and the main DNA methylation marks that have an acknowledged role in gene expression regulation [5‐methylcytosine (5‐mC), 5‐hydroxymethylcytosine (5‐hmC) and N6‐methyladenine (6‐mA)]. Solid lines denote the existence of substantial knowledge on the biological role and/or general patterns of distribution of each one of these epigenetic players, and on how it interacts with environmental contaminants; dashed lines represent a limited understanding; and the absence of a line indicates that no reliable information is available. References: *in vitro* cell models and human‐related models (Jiang *et al*., 2008; Iyer *et al*., 2016; Okamoto *et al*., 2016; Wu *et al*., 2016b; Xiong *et al*., 2017; Miousse *et al*., 2017; Yao *et al*., 2017; Amenyah *et al*., 2020; Cui *et al*., 2022); *Danio rerio* (Hamdi *et al*., 2012; Thomas *et al*., 2013; Kamstra *et al*., 2015; Sanchez *et al*., 2017; Best *et al*., 2018; Zanandrea *et al*., 2020; Torres *et al*., 2021); *Daphnia magna* (Asselman *et al*., 2015, 2017; Kusari *et al*., 2017; Athanasio *et al*., 2018; Lindeman *et al*., 2019); microalgae (Feng *et al*., 2010; Karanthamalai *et al*., 2020; Ferrari *et al*., 2023; Dai *et al*., 2024). Images obtained from BioRender.com.

Despite the growing interest in incorporating epigenetic investigations into (eco)toxicology in recent years, the use of epigenetic endpoints/biomarkers is still somewhat limited. Much remains to be uncovered regarding the effects of several environmental contaminants. In addition, the rapid pace of epigenetics research and technology means that novel insights in the field of environmental epigenetics are continuously emerging. Research efforts are still unevenly distributed across different groups of organisms, with fundamental knowledge concerning the differential epigenetic landscape of several model species used in ecotoxicology studies still limited (Fig. 1). In Fig. 1, we compare knowledge on DNA methylation from *in vitro*‐based models and human‐related models, for which more information is available, with model freshwater organisms relevant in ecotoxicological research and for which more has been done concerning epigenetic responses, based on a dedicated literature search. It becomes clear that although some information exists, fundamental knowledge still is largely lacking for ecotoxicologically relevant models critical for ecological risk assessment frameworks.

Information on epigenetic effects described in (eco)toxicologically relevant species for environmental contaminants listed as priority hazardous substances on Annex X of the European Union Water Framework directive (European Commission, 2013) is summarized in the online Supporting Information (see Table S1). From Table S1 we can conclude that: (*i*) for many of these environmental contaminants there is a lack of knowledge regarding the full spectrum of their epigenetic effects in (eco)toxicologically relevant models; (*ii*) there is little understanding of the underlying mechanisms behind how environmental contaminants cause changes to each epigenetic layer, with the notable exception of cadmium; (*iii*) for many ecotoxicology models there is very limited knowledge concerning epigenetic regulation, particularly for histone modifications and non‐coding RNAs, which makes it hard to ascertain how environmental stressors modulate their epigenetic landscape.

Therefore, the focus herein is to provide a synthetic review of existing knowledge on epigenetic mechanisms with relevance to (eco)toxicology research on animals. We discuss the intricate interplay between the main epigenetic mechanisms and summarize the fundamental differences in epigenetic patterns between vertebrate and invertebrate species. Specific emphasis is given to the underlying mechanisms by which common aquatic environmental contaminants can modulate the epigenome, focusing where possible on invertebrate species. Finally, we highlight future directions that could help guide research towards a better understanding of epigenetic regulation on non‐human models usually used in ecotoxicology. It is hoped that these recommendations will result in better integration of epigenetics into ecotoxicological studies and help to cement epigenetic endpoints as valuable biomarkers for various environmental contaminants. Additionally, integration of epigenetic endpoints with data from other endpoints could provide early molecular initiating events for the Adverse Outcome Pathways (AOPs) frameworks that are increasingly being used in prospective ecological risk assessment.

## II. EPIGENETIC MECHANISMS

There are three principal epigenetic layers or modifications that are capable of gene expression regulation: (*i*) DNA methylation; (*ii*) post‐transcriptional histone modifications; and (*iii*) non‐coding RNAs. Epigenetic alterations to the genome are carried out by specialized enzymes termed ‘writers’, such as DNA methyltransferases (DNMTs) and histone‐modifying enzymes, and ‘readers’, such as proteins containing DNA methyl‐binding domains, chromodomains, bromodomains and Tudor domains, that recognize specific epigenetic marks through their protein‐containing domains and recruit other chromatin modifiers and remodelling proteins (Belzil *et al*., 2016; Biswas & Rao, 2018). All epigenetic marks, i.e. the epigenome, can be reversed by specialized enzymes, often referred to as ‘erasers’. Examples of these enzymes include ten‐eleven translocation (TET) methylcytosine dioxygenases responsible for active/enzymatic DNA demethylation, histone demethylases, histone deacetylases, lysine demethylases, phosphatases and deubiquitylases (Belzil *et al*., 2016; Biswas & Rao, 2018; Greenberg & Bourc'his, 2019). While histone modifications leading to chromatin remodelling are highly reversible, changes in DNA methylation are perceived as more stable and therefore generally understood as long‐term changes to the genome (Belzil *et al*., 2016). Concerning non‐coding RNAs, it is known that they are dynamically expressed in response to both internal and external stimuli, acting as integral parts of epigenetic responses and regulation (Wei *et al*., 2017; Miguel, Lamas & Espinosa‐Diez, 2020). Epigenetic mechanisms are thus globally dynamic and capable of responding to external and internal stimuli, often functioning as the ‘triggers’ for downstream cellular and physiological responses.

### (1) DNA methylation

**Fig. 2:**
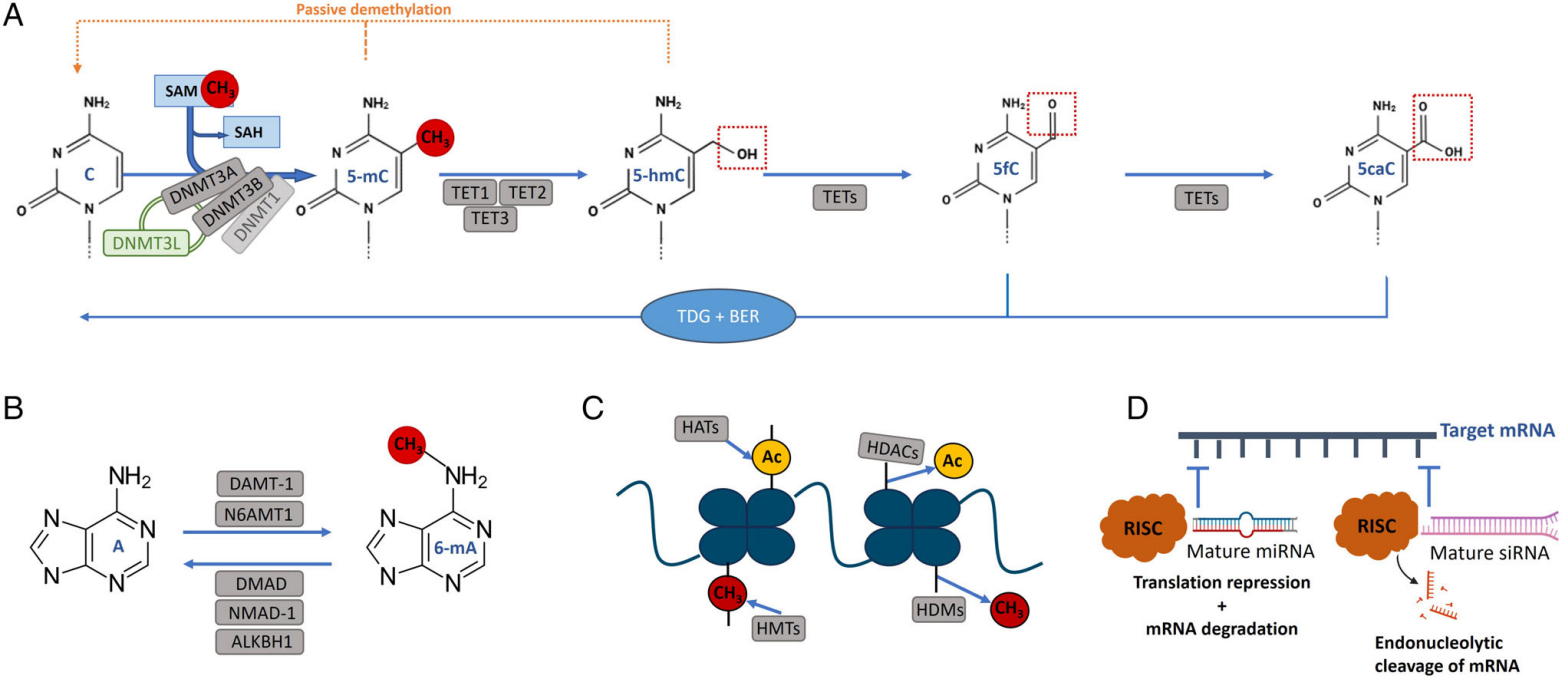
(A) The cytosine methylation cycle and the enzymatic machinery involved: 5‐methylcytosine (5‐mC) formation is catalysed by DNA methyltransferases (DNMTs), with S‐adenosylmethionine (SAM) providing methyl groups and S‐adenosylhomocysteine (SAH) being formed after methyl group transfer to the target cytosine. This 5‐mC state can be passively reversed *via* DNA replication or actively altered in a demethylation process catalysed by ten‐eleven translocation (TET) enzymes that results in 5‐hydroxymethylcytosine (5‐hmC), 5‐formylcytosine (5fC) and 5‐carboxylcytosine (5caC) formation, the latter allowing the return of a modified cytosine to its ‘naked’ form mediated by the thymine DNA glycosylase (TDG)‐dependent base excision repair (BER) pathway. (B) Simplified summary of different ‘writer’ and ‘eraser’ enzymes identified in different species to be involved in adenine methylation. DAMT‐1, DNA N6 adenine methyltransferase 1; DMAD, DNA N6‐methyladenine demethylase; N6AMT1, N6 adenine‐specific DNA methyltransferase 1; NMAD‐1, N6‐methyladenine demethylase 1. (C) Histone tail modifications, in this case methylation and acetylation, that are established by histone methyltransferases (HMTs) and histone acetyltransferases (HATs), then removed by histone demethylases (HDMs) and histone deacetylases (HDACs). (D) Several classes of non‐coding RNAs (ncRNAs) have crucial roles in gene expression regulation. Among them, microRNAs (miRNAs) and small interfering RNAs (siRNAs) play important roles on post‐transcriptional epigenetic regulation of gene expression. Both siRNAs and miRNAs inhibit translation while working in association with a protein complex named RNA‐induced silencing complex (RISC), however they work *via* two different mechanisms. In the case of miRNAs, they often lead to target messenger RNA (mRNA) translational repression and/or degradation, while siRNAs generally cause target mRNA cleavage and degradation. Images obtained from BioRender.com.

DNA methylation involves the covalent addition of a methyl group, usually to the carbon‐5 position of cytosine, to form the fifth base, 5‐methylcytosine (5‐mC), in cytosine–guanine (CpG) dinucleotides (Fig. 2A) (Moore, Le & Fan, 2012; Cheng *et al*., 2012; Belzil *et al*., 2016). Cytosine methylation is widespread in both eukaryotes and prokaryotes (Lewis *et al*., 2020). Genomic regions enriched in 5‐mC are typically highly repetitive and transcriptionally repressed compacted regions of the genome known as heterochromatin (Nishibuchi & Déjardin, 2017). Conversely, genomic regions where 5‐mC is low or absent are typically lightly compacted regions, known as euchromatin, where genes that are more actively transcribed are located (Nishibuchi & Déjardin, 2017). This process, its players and nuances, and its relevance to ecotoxicology are discussed below. A synthesis is provided in Table 1.

**Table 1:** Synthesis on the functional outcomes and features of the best‐known epigenetic mechanism, DNA methylation, and its major players, in eukaryotes, with a focus on non‐human models.

	Role/functional outcome	Additional notes
5‐methylcytosine (5‐mC)	Most common role is gene expression repression, impedes transcription factors binding to DNA, recruits silencing machinery, and/or induces changes to chromatin conformation	Can occur on promoters, enhancers, insulators, gene bodies, intergenic regions and repetitive DNA, leading to different gene expression outcomes; occurs mainly in a CpG context, but can also occur in a CpT and CpA sequence context; is development and tissue specific and plays a crucial role in the cellular differentiation process
	Less common roles include gene expression stimulation by gene body methylation in animals *via* antagonization of Polycomb repressive complex (PRC2), avoiding spurious RNA polymerase II entry and cryptic transcription initiation; gene expression induction by binding of specific transcription factors to methylated DNA in promoter regions or by methylation of silencer or insulator elements in the proximity of promoters	
S‐adenosylmethionine (SAM)	Methyl group donor	Provided by one‐carbon metabolism (folate and methionine); changes in SAM availability and/or in the expression of one‐carbon metabolism‐related genes affect the availability of methyl donors
DNA methyltransferase 1 (DNMT1)	Maintains methylated CpG sites during DNA replication, reinstating original methylation patterns in hemimethylated CpG sites; maintains methylation status once it is established *de novo*	Highly expressed and active during mammalian embryonic development but when cells reach terminal differentiation their expression is much reduced
DNMT3A DNMT3B	*De novo* methylation in originally unmethylated CpG sites	
DNMT3L	Does not catalyse methylation directly, but modulates the activity of DNMT3A and DNMT3B	
DNMT2	Methylates transfer RNA (tRNA) in mammalian genomes; potentially capable of catalysing 5‐mC formation, especially in the context of tRNA–DNA hybrids	
Ten‐eleven translocation methylcytosine translocases (TET1, TET2, TET3)	Conversion of 5‐mC into 5‐hmC, thus generally linked to the restoration of gene expression	Highly expressed and active during mammalian embryonic development and during gametogenesis, where they play a crucial role in epigenetic reprogramming
5‐hydroxymethylcytosine (5‐hmC)	Generally associated with gene expression stimulation, but its exact biological function along with the exact mechanisms through which 5‐hmC induces gene expression are currently unknown	Its distribution is development and age dependent; plays a crucial role in mammalian neurodevelopment and neurological disease development
N6‐methyladenine (6‐mA)	Usually leads to stimulation of gene expression, e.g. activation of transposable elements during embryo development, however its regulatory role is highly variable among species; complementation of temporary 5‐mC loss during epigenetic reprogramming	Its content in eukaryotic genomes is extremely low; is tissue and development specific; occurs mostly in non‐coding regions, particularly in an AG sequence context
DNA N6 adenine methyltransferase 1 (DAMT‐1)	Catalyses adenine methylation	High homology with DNA methyltransferase family A70 enzyme family members
DNA N6‐methyladenine demethylase (DMAD)	6‐mA demethylase in *D. melanogaster*	Belongs to the TET enzyme family, however there are some doubts concerning its role in 6‐mA removal
N6‐methyladenine demethylase 1 (NMAD‐1)	6‐mA demethylase in *C. elegans*	Highly homologous to the ALKB family of enzymes that is responsible for adenine demethylation in the human and mouse genomes

Cytosine methylation is catalysed by DNMTs, with the methyl group donor being S‐adenosylmethionine (SAM) (Fig. 2A) that is provided by one‐carbon metabolism, a complex metabolic network that utilizes nutrients to fuel a variety of metabolic pathways (Poh *et al*., 2016; Mentch & Locasale, 2016; Kusari *et al*., 2017). DNMTs are responsible for establishing and maintaining DNA methylation patterns on cytosines (5‐mC) throughout the genome (Belzil *et al*., 2016). In animal genomes, DNMT1 is a ‘maintenance’ DNMT responsible for maintaining methylated CpG sites during DNA replication and reinstating original methylation patterns in hemimethylated CpG sites (Lyko, 2017); DNMT3A and DNMT3B are responsible for *de novo* methylation (Belzil *et al*., 2016; Lyko, 2017); DNMT3L cannot catalyse DNA methylation directly, but instead modulates the activity of DNMT3A and DNMT3B (Kareta *et al*., 2006; Jia *et al*., 2007); DNMT2 is thought mainly to methylate transfer RNA (tRNA) in mammalian genomes (Schaefer *et al*., 2010; Tuorto *et al*., 2012) but some evidence suggests that this enzyme is also capable of catalysing 5‐mC formation especially in the context of tRNA–DNA hybrids (Hermann, Schmitt & Jeltsch, 2003; Kaiser *et al*., 2017). DNA demethylation occurs either through passive dilution during DNA replication, where DNMTs are inhibited leading to a gradual loss of 5‐mC across successive cell divisions; or by a complex series of chemical reactions catalysed by ‘eraser’ TET enzymes that modify 5‐mC by deamination and/or oxidation reactions (Fig. 2A) (Bochtler, Kolano & Xu, 2017).

DNA methylation (primarily 5‐mC) can occur on promoters, enhancers, insulators, gene bodies, intergenic regions and repetitive DNA, defining changes to gene expression (Jones, 2012; Varriale, 2014). Historically, CpG methylation in the promoter region of vertebrate genes has been associated with loss of gene expression and found to be development and tissue specific (Moore *et al*., 2012; Lokk *et al*., 2014; Suelves *et al*., 2016). However, it is now known that the function of DNA cytosine methylation seems to vary with context and genomic location of the methylated CpG dinucleotides, repressing or stimulating gene expression (Jones, 2012; Varley *et al*., 2013; Greenberg & Bourc'his, 2019). Accordingly, highly transcribed genes have been found to have abundant intragenic or gene body methylation in animal genomes, which aids in transcription initiation and elongation *via* complex DNA methylation–gene transcription machinery and DNA methylation–transcription factors interactions (Wu *et al*., 2010; Jones, 2012; Buck‐Koehntop & Defossez, 2013; Neri *et al*., 2017; Vogt, 2021). Additionally, methylation of silencer or insulator elements, found in varying proximity of the promotor regions of genes, leads to the abolishment of their repressive activities on gene expression (Jones & Takai, 2001; Angeloni & Bogdanovic, 2019).

Although 5‐hydroxymethylcytosine (5‐hmC) was previously thought to represent only an intermediate state between methylated and unmethylated cytosines (Fig. 2A), there is now evidence that it also acts as a stable epigenetic marker essential in neurodevelopment. Fluctuations in its distribution are known to contribute to neurological disease development in mammals (Song *et al*., 2011; Sun *et al*., 2014; Belzil *et al*., 2016). In mammals, three ‘eraser’ enzymes TET1, TET2, and TET3 are responsible for 5‐mC conversion to 5‐hmC, a crucial mechanism for restoring gene expression (Kohli & Zhang, 2013; Sun *et al*., 2014). Generally, 5‐hmC enrichment is thought to be associated with increased gene expression (Colquitt *et al*., 2013; Sun *et al*., 2014).

Beyond the classical assumption that DNA methylation corresponds to the methylation of cytosines (5‐mC), DNA bases other than cytosine can undergo epigenetic modifications. *Caenorhabditis elegans* and *Drosophila melanogaster* have no or negligible amounts of 5‐mC or 5‐hmC but do have detectable levels of N6‐methyladenine (6‐mA) in 0.3% and 0.0002–0.07% of total adenines, respectively (Greer *et al*., 2015; Zhang *et al*., 2015; Kumar, Chinnusamy & Mohapatra, 2018; Kong *et al*., 2022). In *C. elegans*, 6‐mA seems to play a role in stimulating gene expression across most of the life of the animal (Greer *et al*., 2015), while in *D. melanogaster* it is thought to be associated mainly with transposable elements activation during embryonic development as well as with enforcing tissue‐ and development‐specific gene expression control in adult flies (Zhang *et al*., 2015; Shah, Cao & Ellison, 2019). However, for *Drosophila melanogaster* doubts remain concerning the *de facto* functional role of 6‐mA, with recent work suggesting that this epigenetic mark may have a more limited gene regulation role than previously reported (Boulet *et al*., 2023). In zebrafish (*Danio rerio*), trace amounts of 6‐mA (around 0.1–0.2% of total adenines) regulate transposable elements expression during early embryonic development (Liu *et al*., 2016). There is also evidence for a link between 6‐mA and expression regulation of non‐coding regions in *Xenopus laevis* genome (Koziol *et al*., 2015). In some eukaryotes, 6‐mA seems to be depleted in coding regions, especially exons, and mainly exists in an AG sequence context in *C. elegans*, zebrafish, *Xenopus laevis* and *Mus musculus* (Greer *et al*., 2015; Koziol *et al*., 2015; Liu *et al*., 2016). Despite 6‐mA being a well‐studied prokaryotic epigenetic DNA modification, the available information in eukaryotes is still insufficient to understand clearly the dynamics and evolutionary consequences of adenine methylation. Existing evidence suggests that: (*i*) 6‐mA content is extremely low in eukaryotic genomes, with a species‐specific distribution; (*ii*) adenine methylation likely occurs in a developmentally regulated manner, playing a crucial role during embryonic development in some eukaryotic species (Zhang *et al*., 2015); and (*iii*) there are marked differences in the regulatory role of 6‐mA across eukaryotes (Koziol *et al*., 2015; Iyer, Zhang & Aravind, 2016; Liu *et al*., 2016). Intriguingly, an association between 5‐mC reprogramming (erasure of methylation marks) and an accumulation of 6‐mA has been found in *Danio rerio* and *Sus domesticus* (Luo & He, 2017). Similar to 5‐mC, there are ‘writer’ and ‘eraser’ enzymes responsible for establishing and removing 6‐mA marks from the DNA, respectively (Fig. 2B). A putative DNA 6‐mA methyltransferase, DNA N6 adenine methyltransferase 1 (DAMT‐1), was first reported in *C. elegans* and it shares high homology with members of the A70 family of DNMTs (Greer *et al*., 2015). In other species there is only limited information available, with the identity of these enzymes requiring further characterization and confirmation. Focusing on ‘eraser’ enzymes, often called 6‐mA demethylases, the first proposed candidate DNA 6‐mA demethylase were described in *D. melanogaster* (DMAD, DNA N6‐methyladenine demethylase) (Zhang *et al*., 2015) and in *C. elegans* (NMAD‐1, N6‐methyladenine demethylase 1) (Greer *et al*., 2015). Generally, two families of enzymes are capable of demethylating adenines, the ALKB family (to which NMAD‐1 belongs), and the related, but distinct, TET family (to which DMAD belongs) (Zhang *et al*., 2015; Yao *et al*., 2018; Boulias & Greer, 2022). There remain reasonable doubts concerning the role of TET/DMAD enzymes in the removal of 6‐mA marks from DNA (Iyer *et al*., 2016; Boulet *et al*., 2023). The preliminary knowledge available concerning the presence of potential 6‐mA methyltransferases and demethylases suggests that 6‐mA could be actively regulated, thus being a DNA epigenetic mark in its own right.

### (2) Histone modifications

Histone modifications are also an important mechanism of epigenetic regulation (Fig. 2C), which relates to their role in defining chromatin structure (Strahl & Allis, 2000; Cheng *et al*., 2012). In eukaryotes, there is a group of highly conserved histone proteins (H3, H4, H2A, H2B and H1) which in their N‐terminal tails can contain a variety of post‐translational modifications, such as acetylation, methylation, phosphorylation, ADP‐ribosylation, ubiquitination and SUMOylation (Suganuma & Workman, 2008; Cheng *et al*., 2012). In most organisms, histone acetylation and phosphorylation stimulate gene expression. The acetylation of lysine residues on histone tails leads to accessible chromatin because it prevents chromatin from folding into higher‐order structures (Li & Reinberg, 2011). Histone methylation and SUMOylation mostly represses gene expression by obstructing accessibility to transcriptional regulators and other essential machinery (Kouzarides, 2007; Vogt, 2021). However, histone methylation has been found to be implicated in both activation and repression of transcription (Kouzarides, 2007; Karlić *et al*., 2010).

Histone tail modifications are dynamic marks regulated by the activity of several specialized enzymes like histone acetyltransferases, histone methyltransferases, protein arginine methyltransferases and kinases (Fig. 2C) (Belzil *et al*., 2016; Vogt, 2021). Modified histones form a recognizable ‘code’ that often leads to transcriptional regulation *via* changes in chromatin structure and accessibility (Strahl & Allis, 2000; Vogt, 2021), but also act as ‘receptors’ for regulatory proteins that mediate downstream functions (Strahl & Allis, 2000; Cheng *et al*., 2012). Interestingly, contradictory histone tail modifications can lead to chromatin regions containing both activating and repressing modifications, referred to as bivalent chromatin domains, which are able to regulate gene expression more rapidly by allowing genes present in these regions to be promptly activated (Bernstein *et al*., 2006; Harikumar & Meshorer, 2015). Another less widely known impact of histone modifications and consequent changes in chromatin structure is that they also regulate access to DNA by repair enzymes, which has an influence on the frequency of DNA break repair (Shen & Laird, 2013).

### (3) Non‐coding RNAs

Non‐coding RNAs (ncRNAs) are also epigenetic modulators of gene expression (Fig. 2D), that can be of two types: long ncRNAs and small ncRNAs (Zhang *et al*., 2021). Long ncRNAs include large intergenic ncRNAs, circular RNAs, non‐circular competitive endogenous RNAs and enhancer RNAs (Palazzo & Lee, 2015), while small regulatory ncRNAs encompass microRNAs (miRNAs), small interfering RNAs (siRNAs), small nucleolar RNAs, and PIWI‐interacting RNAs (Piwi‐RNAs). Indeed, RNA not only functions as a simple messenger between DNA and proteins, but also has a crucial role in gene expression regulation and on genome organization (Morris & Mattick, 2014). Regulatory ncRNAs play central roles in transcriptional and post‐transcriptional epigenetic processes, while their own transcription is also under epigenetic control (Morris & Mattick, 2014).

Small ncRNA‐mediated regulation of gene expression encompasses two main mechanisms, translational repression and messenger RNA (mRNA) degradation (by miRNAs) and mRNA cleavage (by siRNAs) (Hutvagner & Zamore, 2002; Moore *et al*., 2012). The functions of miRNAs and siRNAs overlap with each other to a certain extent, with the major difference between them being that the latter inhibits the expression of a specific target mRNA, while the former regulates the expression of multiple mRNAs. Additionally, small ncRNAs influence gene expression by recruiting chromatin‐modifying complexes and DNMTs to a particular genomic locus, leading to transcriptional repression of the genes located at these genomic regions (Mattick, 2007). Alongside their ability to recruit DNMTs, miRNAs may indirectly regulate DNMTs expression: the loss of miRNA‐290 in mouse embryonic stem cells leads to reduced DNMT3A and DNMT3B expression and therefore loss of *de novo* DNA methylation (Sinkkonen *et al*., 2008). Also, siRNAs are capable of modulating histone and/or DNA methylation to repress transcription *via* RNA interference pathways (Holoch & Moazed, 2015).

Piwi‐RNAs are a family of single‐stranded RNAs associated with PIWI proteins expressed mainly in germline cells, and capable of silencing coding regions of the genome, particularly transposable elements and virus‐derived nucleic acids (Reuter *et al*., 2011; Ernst, Odom & Kutter, 2017; Pinto *et al*., 2022). Specifically, Piwi‐RNAs can promote the generation of heterochromatic marks, such as repressive histone modification H3K9me3, around the target sequences. The latter often are transposable elements and/or facilitate the degradation of target mRNAs by serving as guides for specialized protein complexes. Interestingly, due to the architecture of their biogenesis pathway, Piwi‐RNAs provide an adaptive, sequence‐based immunity to rapidly evolving transposons and viruses, being at the same time capable of regulating highly conserved host genes (Hess *et al*., 2011; Ozata *et al*., 2018). Piwi‐RNAs are highly conserved across animal species, further substantiating their important biological roles (Grimson *et al*., 2008). For example, in *C. elegans* Piwi‐RNAs have an important role in transgenerationally inherited transcriptional responses to external stress (Moore, Kaletsky & Murphy, 2019), and in an intricate interaction between Piwi‐RNAs and siRNAs that is responsible for the epigenetic silencing of genomic regions linked with neurobehavioural regulation in germline cells (Casier *et al*., 2019).

As for long ncRNAs, since they overlap with or are interspersed between several coding and non‐coding transcript variants, they are mostly associated with the regulation of expression of neighbouring protein‐coding genes, often in a tissue‐ and developmental‐stage specific manner (Lopez‐Pajares, 2016). One of the best‐known mechanisms by which long ncRNAs are capable of regulating gene expression occurs in the X chromosome of the mouse, where a ncRNA named long X‐inactive specific transcript recruits and targets the Polycomb repressive complex 2 (PRC2) (Zhao *et al*., 2008; Lopez‐Pajares, 2016). PRC2 is a chromatin‐silencing protein complex responsible for maintaining X‐chromosome inactivation, *via* deposition of the repressive H3K27me3 histone mark (Zhao *et al*., 2008; Lopez‐Pajares, 2016). Long ncRNAs are then capable of repressing and inducing gene expression in *cis*, mainly through recruitment of activating and repressive histone remodelling complexes (Lopez‐Pajares, 2016). Nuclear long ncRNAs also have also a role in altering the phosphorylation status of serine/arginine splicing factors, thereby having the ability to modulate alternative splicing of coding gene precursor mRNA (pre‐mRNAs) (Tripathi *et al*., 2010; Lopez‐Pajares, 2016). Cytoplasmatic long ncRNAs are also involved in the regulation of mRNA stability *via* recruitment of RNA binding proteins either to promote or prevent mRNA decay and therefore regulate post‐transcriptional gene expression (Kretz *et al*., 2013). Another type of long ncRNAs are circular RNAs. These looped RNA molecules are derived from various regions of the genome, preferentially expressed in neural tissues, and can regulate miRNAs (and other molecules) by functioning as ‘molecular sponges’, effectively inhibiting miRNAs suppression of mRNAs (Hansen *et al*., 2013; Cortés‐López & Miura, 2016; Meng *et al*., 2019). Enhancer RNAs are another class of long ncRNAs that are transcribed from active enhancer regions that are capable of modulating chromatin accessibility to cognate promoters. Therefore, they facilitate enhancer–promoter interactions, transcription factor entrapment on genomic sites and transcriptional machinery recruitment, ultimately leading to upregulated target gene expression (Lewis, Li & Franco, 2019; Sartorelli & Lauberth, 2020).

## III. THE DYNAMIC INTERPLAY AMONG EPIGENETIC MODIFICATIONS

Although existing ecotoxicological studies have focused mostly on one or the other epigenetic mechanism, it is important to point out evidence from studies using human‐related model systems (i.e. *in vitro* cell lines and/or human health‐related model organisms) indicating that there is extensive crosstalk among the three main epigenetic mechanisms and that independent regulatory action is rare. In mammals, DNA methylation is highly interconnected to post‐transcriptional changes at histone lysine residues, with each epigenetic system mechanistically relying on the other for normal regulation of chromatin conformation (Chen, 2011; Rose & Klose, 2014). As such, histone modification can direct DNA methylation patterns and in turn, DNA methylation can serve as a template for histone modifications (Rose & Klose, 2014; Du *et al*., 2015). It is known that DNMTs directly interact with enzymes that regulate histone modifications in genomes ranging from fungi to humans. Here, both DNMT1 and DNMT3A are known to bind to the histone methyltransferase SUV39H1 that restricts gene expression by promoting methylation on histone 3 at lysine 9 (H3K9), therefore maintaining stable repressive conditions for gene expression (Fuks *et al*., 2003; Du *et al*., 2015). In mammals, DNMT3L also binds to trimethylated histone 3 tails leading to an increase in DNMT3L levels and the recruitment of DNMT3A and DNMT3B to methylate DNA (Li & Reinberg, 2011; Janssen & Lorincz, 2022). H3K4 trimethylation impairs the binding of DNMTs to histone 3 tails and therefore prevents methylation, maintaining a permissive chromatin state (Torres & Fujimori, 2015). The combined effects of histone modifications and DNA methylation are thought to occur most often in genes or genomic regions that are subjected to more dynamic variations in gene transcription, such as critical genes for brain function (Bagot & Meaney, 2010). Experimental data gathered from *C. elegans* suggest a functional correlation between 6‐mA DNA methylation marks and H3K4 methylation (H3K4me2), an association that seems to be essential to stable transmission of epigenetic information across generations in this nematode (Greer *et al*., 2015).

Another example of the extensive crosstalk among epigenetic mechanisms is the positive feedback mechanisms between small ncRNAs amplification and histone modification or DNA methylation, which is responsible for maintaining stability of repressive epigenetic conditions in several species (Holoch & Moazed, 2015; Belzil *et al*., 2016). In these feedback mechanisms, small ncRNAs work as vectors between genes and environment cues and as essential parts of complexes responsible for chromatin ‘silencing’ (Zofall & Grewal, 2006; Gapp *et al*., 2014). Curiously, these epigenetic loops are key players in epigenetic inheritance of histone and DNA methylation patterns, specifically in *M. musculus* and *C. elegans* germlines, where small ncRNAs act as heritable epigenetic fingerprints for internal or environmentally induced alterations that maintain gene silencing across several generations (Gu *et al*., 2012; Gapp *et al*., 2014; Rechavi *et al*., 2014). *C. elegans* was shown to possess an interesting complex epigenetic mechanism of regulation involving ncRNAs and heritable histone modifications (Gu *et al*., 2012). In *D. melanogaster*, the existence of a self‐reinforcing loop between Piwi‐RNAs biogenesis and the maintenance of repressive histone H3 lysine 9 (H3K9) methylation marks has been reported (Holoch & Moazed, 2015).

## IV. INTERSPECIFIC VARIATION OF EPIGENETIC MECHANISMS

Due to growing concerns regarding bioethics and to avoid unnecessary suffering to vertebrate models, the majority of (eco)toxicological testing is now performed using invertebrate species (Doke & Dhawale, 2015). Additionally, many of these invertebrates have a wide distribution, accessibility, easy maintenance, handling, and several reproduce asexually, allowing separation of epigenetic variation from genetic variability. These features make them promising models for epigenetic studies and for the development of epigenetic biomarkers (Jeremias *et al*., 2020).

The epigenetic machinery and its characteristics are known to vary among species. This is particularly challenging when using ecologically relevant models in (eco)toxicoepigenetic studies, where there is limited genomic information (genomes are much more sparsely annotated than in human‐related model systems) and where epigenetics mechanisms and their crosstalk have not been fully described. This subsection does not constitute a complete list of all the differences in epigenetic patterns between vertebrates and invertebrate species, nor does it intend to enumerate the epigenetic characteristics of a particular ecotoxicology model. Rather, its objective is to illustrate the vast interspecific diversity of epigenetic patterns that exist (with an emphasis on differences between vertebrates and invertebrates), focusing on 5‐mC DNA methylation where more fundamental knowledge is available. Importantly, a major goal is to raise awareness of the multiple factors that should be considered when selecting a suitable model organism for (eco)toxicoepigenetic studies and when comparing epigenetic responses between non‐related species. Indeed, DNA methylation occurs in most animals (Vogt, 2021) but fundamental differences exist concerning its distribution throughout the genome, the percentage of DNA that can be methylated, the nucleotides that are methylated and the sequence context where methylation occurs.

Although the mechanisms of epigenetic regulation and genome‐wide DNA methylation patterns are relatively well conserved across many vertebrate species (Goll & Halpern, 2011; Zhou *et al*., 2017), the same is not true concerning invertebrates (Zemach *et al*., 2010). For example, 5‐mC patterns on gene bodies seem to have higher conservation between vertebrates and invertebrates with methylation of promoters and repetitive sequences displaying more variability, potentially indicating divergent evolution of the regulatory role of DNA methylation across the vertebrate–invertebrate boundary (Jones, 2012; Greenberg & Bourc'his, 2019; Vogt, 2021).

In mammals and other vertebrates, DNA methylation often functions as a repressive transcriptional mark that mostly occurs on cytosines in a CpG context and follows a genome‐wide methylation pattern, with high percentages of methylated DNA in somatic cells (60–90% of all CpG dinucleotides) (Jones, 2012; Li & Zhang, 2014; Vogt, 2021). Interestingly, fish tend to have higher percentages of global DNA methylation than mammals (Goll & Halpern, 2011; Head, 2014). DNA methylation in vertebrates mostly occurs in CpG dinucleotides located in intergenic regions, namely on repetitive DNA (satellite DNA and transposable elements located inside and outside genes), and at lower frequency in gene introns and exons (Ghosh *et al*., 2010; Li & Zhang, 2014). By contrast, CpG islands that are mostly found close to promoters of approximately two‐thirds of all genes usually remain unmethylated in the germline and in most adult somatic tissues (Ghosh *et al*., 2010; Li & Zhang, 2014; Head, 2014; Hernando‐Herraez *et al*., 2015). Methylation of CpG islands located in gene promotors of vertebrate genomes has been associated to the silencing of genes on the inactive X chromosome, silencing of imprinted genes and tissue‐specific gene expression regulation (Jones & Takai, 2001; Greenberg & Bourc'his, 2019).

Invertebrates show low percentages of DNA methylation, with some species having less than 1% of all CpG dinucleotides methylated (Takayama *et al*., 2014; Trijau *et al*., 2018; Vogt, 2021). DNA methylation in invertebrate genomes follows a mosaic pattern of distribution, consisting of sections of methylated DNA (usually gene bodies, especially exons) that are interspersed with unmethylated regions (normally intergenic regions) (Trijau *et al*., 2018; Vogt, 2021). Generally, DNA methylation in invertebrate species occurs mostly in gene bodies, associated with transcriptional enhancement (Suzuki *et al*., 2007; Kvist *et al*., 2018; Glastad, Hunt & Goodisman, 2019), while methylation of intergenic repetitive sequences, such as transposable elements and tandem repeats, is low or mostly absent (Zemach *et al*., 2010; Lewis *et al*., 2020; Chaturvedi *et al*., 2023). In fact, the regulatory function of gene body methylation is evolutionarily ancient and highly conserved between vertebrates and invertebrates, since stably expressed genes exhibit high percentages of methylation within gene bodies, particularly in exonic regions, across animal genomes (Zemach *et al*., 2010; Kvist *et al*., 2018). Gene body methylation in invertebrates seems to accumulate in highly conserved essential genes (e.g. housekeeping genes), while genes with tissue‐specific expression are poorly methylated (Suzuki *et al*., 2007; Kvist *et al*., 2018). Curiously, in *Daphnia* sp., a widely used invertebrate model in ecotoxicology studies, gene body methylation occurs mostly on the first exons of coding genes, strongly correlating with enhanced gene expression. As such, it likely plays a role in preventing transcriptional interference and in alternative splicing modulation (Asselman *et al*., 2017; Kvist *et al*., 2018; Hearn, Plenderleith & Little, 2021; Chaturvedi *et al*., 2023). A similar scenario might also occur in the Pacific oyster (*Crassostrea gigas*), where the most methylated exons are more prone to be integrated in mature gene transcripts, indicating a potential regulatory role of gene body methylation in alternative splicing (Olson & Roberts, 2014; Song, Li & Zhang, 2017). Another interesting characteristic found in *D. magna* is that gene body cytosine methylation seems to occur at a higher percentage within serine and threonine codons of ribosomal genes under stress conditions, which may indicate that methylation in specific amino acids can be an adaptative mechanism employed by *D. magna* to produce alternative or more stable proteins, *via* alterations to alternative splicing patterns (Asselman *et al*., 2017).

In invertebrates, gene promoters are generally poor in CpG dinucleotides and usually are not methylated (Zemach *et al*., 2010; Kvist *et al*., 2018; Lewis *et al*., 2020; Klughammer *et al*., 2023); caution is needed in interpreting this finding given the sometimes quite fragmented genome assemblies available and poorly characterized promoter regions. However, some invertebrate species were found to have methylated gene promotors (Rivière, 2014; Keller, Han & Yi, 2016), but doubts still exist as to whether if this characteristic was independently acquired by these species or if it has an ancestral evolutionary origin, and on the functional roles that promoter methylation has independently of gene body methylation (Keller *et al*., 2016).

Concerning introns in invertebrate genomes, the most common view is that some species, such as *D. magna*, exhibit a near zero intron methylation landscape (Asselman *et al*., 2017; Trijau *et al*., 2018; Kvist *et al*., 2018), while others (for example, the honeybee *Apis mellifera*) have significantly lower DNA methylation in introns when compared to exons, with some accumulation of 5‐mC at exon–intron boundaries (Jeong *et al*., 2018). The general intron hypomethylation in invertebrates is a marked difference to vertebrates, where these genomic regions are normally highly methylated apart from the first introns of highly transcribed genes (Kvist *et al*., 2018). Some plausible explanations exist for this difference, including: (*i*) vertebrate genomes exhibit large intronic sequences and a higher number of these regions (Olthof, White & Kanadia, 2022), while in invertebrates these regions are usually smaller (Schwartz *et al*., 2008; Wu *et al*., 2013), possibly leading to a requirement for more methylation in introns of vertebrate genomes to regulate them better; (*ii*) different exon–intron structure and subsequent differences in splicing regulation between vertebrates and invertebrates (Gelfman *et al*., 2012; Lev Maor, Yearim & Ast, 2015); and (*iii*) the high number of transposable elements in intronic regions of vertebrate genomes, especially among mammals, compared to the relatively lower presence of these repetitive elements in invertebrate introns (Sela, Kim & Ast, 2010), as their expression is controlled *via* DNA methylation (Ying *et al*., 2022; Pinto *et al*., 2022).

Much remains to be understood concerning the complex DNA methylation landscape in invertebrate genomes, but clear differences exist compared to vertebrates as well as among invertebrate species, thus interspecific interpretations should be made very carefully. While some invertebrates have almost undetectable levels of DNA methylation, others have high percentages of methylated DNA (Trijau *et al*., 2018; Kvist *et al*., 2018; de Mendoza *et al*., 2019; Klughammer *et al*., 2023). Variation in conservation of DNMT coding genes among invertebrate genomes, with some species only having one DNMT (Hendrich & Tweedie, 2003; Rošić *et al*., 2018) and others having a full set of DNMTs (Schaefer & Lyko, 2007; Hearn *et al*., 2021), is also frequent. For example, some nematode species have the full set of DNMTs (e.g. *Romanomermis culicivorax*, which has both DNMT1 and DNMT3) and show much higher percentages of 5‐mC than other nematode species with a single DNMT (e.g. *Nippostrongylus brasiliensis*, which has only DNMT2) where 5‐mC levels are near‐negligible (Rošić *et al*., 2018). The presence, pattern and regulatory role of DNA methylation in DNMT2‐only genomes is not fully resolved (Raddatz *et al*., 2013) and alternative explanations for the variability of the DNA methylation landscape in invertebrates exist. One suggests that some invertebrate species have a vertebrate‐like DNA methylation‐based defence mechanism against intergenic and gene body transposable elements, resulting in higher percentages and broader genome distribution of methylated DNA (de Mendoza *et al*., 2019; Ying *et al*., 2022). By contrast, other invertebrate species have most intergenic repetitive sequences unmethylated (Zemach *et al*., 2010; Gatzmann *et al*., 2018; Chaturvedi *et al*., 2023) with a limited number of transposable elements located in methylated introns, possibly as a side effect of the characteristic extensive gene body methylation (Gatzmann *et al*., 2018; de Mendoza, Lister & Bogdanovic, 2020). Differences concerning promoter and intron methylation levels can also explain the differences in total percentage of methylated DNA and its variable distribution patterns among invertebrate species. Some invertebrate species evolutionarily closer to vertebrates, like the tunicate *Ciona intestinalis*, have intronic regions near transposable elements insertions with considerable amounts of methylated DNA, possibly as a response to invasion of intronic regions by transposable elements (Keller *et al*., 2016) as in vertebrates. A similar situation is found in the *Hydra vulgaris* genome, where intronic transposable elements are preferentially targeted by DNA methylation leading to a high percentage of intronic DNA methylation (Ying *et al*., 2022).

Some invertebrate species are thought to have lost 5‐mC in a CpG context altogether, for example *D. melanogaster*; in this species, it seems that the very low percentage of methylated cytosines (0.034% of total cytosines) occur in an CpT and CpA context, especially during early life stages (Lyko, Ramsahoye & Jaenisch, 2000; Capuano *et al*., 2014; Takayama *et al*., 2014; Lewis *et al*., 2020). The different sequence context where DNA methylation takes place in *D. melanogaster* can be explained by the fact that this fly species only has a single DNMT gene, DNMT2 (Vieira *et al*., 2018), which was found to have specificity for cytosine methylation in a CpT/A sequence context (Kunert *et al*., 2003). Despite these findings, the regulatory functions of 5‐mC in *D. melanogaster* remain largely unknown, and subject to debate (Raddatz *et al*., 2013; Takayama *et al*., 2014). Other invertebrate species do not have detectable levels of 5‐mC and DNA methylation occurs on different nucleotides (Greer *et al*., 2015). For example, in *C. elegans* DNA methylation has been lost in cytosines and mostly occurs in adenines (Greer *et al*., 2015). This nematode indeed does not encode homologs of any of the ‘traditional’ DNMTs responsible for cytosine methylation (Wenzel, Palladino & Jedrusik‐Bode, 2011; Rošić *et al*., 2018). As previously discussed (Section II.1), it does however encode writer and eraser enzymes capable of catalysing adenine methylation and demethylation, respectively (Greer *et al*., 2015). In contrast to 5‐mC, there is very limited and conflicting knowledge available concerning 6‐mA (see Section II.1), precluding any assessment of differences between vertebrates and invertebrates. Additionally, it would be of interest to delineate the similarities and differences between the mechanistic roles of 6‐mA and 5‐mC across eukaryotic genomes, focusing on the vertebrate/invertebrate boundary.

More fundamental knowledge is also required regarding 5‐hmC, to assess the similarities and differences concerning this epigenetic mark between vertebrate and invertebrate species. In human and mouse cerebellum tissue, 5‐hmC was found to occur mainly inside gene bodies near critical gene regulatory motifs such as transcription start and termination sites, suggesting an important role in transcription regulation (Song *et al*., 2011; Kato & Iwamoto, 2014). Current knowledge seems to indicate similar biogenesis mechanisms and regulatory roles for this epigenetic mark in invertebrate species, where it is responsible for development‐specific expression regulation of developmental genes and is associated with high chromatin‐accessibility states (Wojciechowski *et al*., 2014; Skvortsova *et al*., 2022). Despite further confirmation being required, it seems that 5‐hmC regulatory function is conserved across eukaryotic genomes (Wojciechowski *et al*., 2014; Skvortsova *et al*., 2022).

Concerning histone modifications, much less information regarding the similarities or differences between vertebrates and invertebrates is available. In general, most histone modifications and associated mechanisms are relatively conserved between vertebrate and invertebrate species, especially concerning histone modification occurring in promotor and enhancer regions (Schwaiger *et al*., 2014; Dattani *et al*., 2018; Lee *et al*., 2020). The transcriptional outcomes of histone modifications depend on several parameters including number of methyl groups, the residue bearing the modification, gene and cell types, genome organization and chromatin context (Zhou, Goren & Bernstein, 2010; Vastenhouw & Schier, 2012). This means that the same histone modification can have different regulatory functions among organisms (Spada, Vincent & Thompson, 2005). Moreover, the limited data available seem to suggest that targeting mechanisms of histone‐modifying enzymes are conserved among animal species, but the regulatory outcome of these modifications might not always be the same.

As for ncRNAs, miRNAs sequences seem to be highly conserved between *C. elegans*, where they were first discovered, and vertebrate genomes (Pasquinelli *et al*., 2000; Qu & Adelson, 2012). The same seems to be true for some long ncRNAs, which have expression patterns and functionality similarities across vertebrate genomes, suggesting conserved functions (Hezroni *et al*., 2015; Yu, Zhao & Li, 2016). From the existing works concerning the biological role of long ncRNAs, one can conclude that these are important players in gene expression regulation during development, and in regulating stress responses by critical genes both in vertebrate and invertebrate species (Li *et al*., 2012; Nam & Bartel, 2012; Détrée *et al*., 2017). This apparent conservation of some ncRNAs across the vertebrate–invertebrate boundary could make ncRNAs valuable biomarkers for evaluating epigenetic toxicity across different species. However, beyond occasional claims and very limited evidence on invertebrate species, there is a lack of fundamental studies allowing a better understanding of this possibility.

Epigenetic reprogramming (the reset of most of the epigenetic marks) is thought to be limited or not to occur completely in several invertebrate species (Xu *et al*., 2019; Liew *et al*., 2020; Yagound *et al*., 2020). But this is very variable among species: starfish, sea urchins and sea squirts have limited epigenetic reprogramming in specific genomic regions; bumblebees show a gender‐specific erasure of epigenetic marks (Hunt *et al*., 2024); cnidarians and honeybees do not have detectable or yet characterized epigenetic reprogramming process (Xu *et al*., 2019; Yagound *et al*., 2020). By contrast, in mammalian and other vertebrate genomes genome‐wide epigenetic reprogramming occurs in two major waves: during gametogenesis in primordial germ cells and after fertilization before implantation. Only a limited number of genes and repetitive DNA escape this mechanism, namely parental imprinted genes and some families of transposable elements (Head, 2014; Monk, 2015). When implantation occurs, DNA methylation levels are restored by *de novo* methylation, triggering cell lineage differentiation. The limited epigenetic reprogramming in invertebrates has important repercussions regarding the heritability of epigenomic modifications in these species, potentially enhancing their role as good models to study transgenerational epigenetic marks (Jeremias *et al*., 2018, 2022; de Mendoza *et al*., 2020).

## V. MECHANISMS BEHIND EPIGENOME CHANGES INDUCED BY ENVIRONMENTAL CONTAMINANTS: THREE EXPLORABLE CASE STUDIES

Regardless of the growing interest in understanding the epigenetic alterations induced by environmental contaminants, the mechanistic links between exposure and altered epigenetic status have not been fully established. These links are critical to explore epigenomics efficiently within ecological risk assessment frameworks. Environmental compounds can have a direct or indirect impact on the activity of epigenetic modifying enzymes, modulate the expression of genes responsible for encoding essential epigenetic machinery, or interfere with the availability of methyl donors in the cell (through interference in the methionine cycle), among other mechanisms. Beyond this wide range of mechanisms, there are also conflicting pathways that may constrain the definition of clear epigenetic effects of a given environmental contaminant. In this section, we move towards an improved mechanistic understanding of these processes to offer a better understanding of how epigenome changes occur following exposure to three environmental contaminants: cadmium (focusing on DNA methylation), arsenic (focusing on histone modifications) and chromium (focusing on ncRNAs). These are legacy environmental contaminants for which there is appreciable evidence available not restricted to human models. Due to the similar mechanisms of toxic action in ecotoxicological models for these three contaminants, integration of information gathered from exposures to each can potentially be extended to the others towards a better mechanistic understanding of their epigenetic toxicity. Assisting the discussion below, Table 2 provides a summary of existing evidence and some critical details to facilitate interpretation.

**Table 2:** Summary of the main mechanisms through which environmental contaminants can influence epigenetic mechanisms. Three legacy aquatic contaminants for which information exists concerning the mechanisms behind their modulation of the epigenome are included: cadmium (Cd) to illustrate the mechanisms behind DNA methylation modulation; arsenic (As) to complement information on DNA methylation and for mechanisms behind histone modification responses; and chromium (Cr) for ncRNAs. Additional links could potentially be made for each chemical and epigenetic mechanism combination, but for the sake of clarity and brevity are omitted here. Information on the mechanistic links between environmental contaminant exposure and epigenome modulation is retrieved from ecotoxicologically relevant species wherever possible. 5‐hmC, 5‐hydroxymethylcytosine; 5‐mC, 5‐methylcytosine; 6‐mA, N6‐methyladenine; DNMT, DNA methyltransferase; miRNA, microRNA; ncRNA, non‐coding RNA; SAM, S‐adenosylmethionine; TET, ten‐eleven translocation methylcytosine dioxygenase.

	Monitored change	Mechanistic notes	Epigenetic effect
**Cd**	↓ DNMT activity (acute exposure)	Cd binds to the DNA binding site of the enzyme, leading to inhibition that is non‐reversible with increasing substrate concentration (Takiguchi *et al*., 2003) Oxidation of DNMTs affects DNA binding capacity (Franco *et al*., 2008)	↓ DNA methylation
	↑ DNMT activity (chronic exposure)	Occurs *via* a compensatory mechanism leading to increased *dnmt* transcription, after initial inhibition of DNMTs activity (Jiang *et al*., 2008) Occurs *via* recruitment for DNA repair after oxidative‐damage‐caused DNA strand breaks (Hayashi *et al*., 2019; Jiang *et al*., 2020)	↑ DNA methylation
	↑ 5‐mC to 5‐hmC	Passive conversion under oxidative stress conditions (Belzil *et al*., 2016)	↓ DNA methylation
	↓ Ability of DNA to serve as a suitable substrate for writer enzymes	DNA damage, mutations, and chromosomal alterations following oxidative stress may indirectly lead to methylome remodelling (Pogribny *et al*., 2005; Tunc & Tremellen, 2009)	↓ DNA methylation
**Cd** **As**	↓ TET activity	Cd and As cause direct inhibition of TET activity due to interference with TET zinc finger domains (Xiong *et al*., 2017; Wang *et al*., 2021; Keith *et al*., 2021)	↑ DNA methylation
	↓ SAM methyl donor	**Cd** leads to an increased need for reduced glutathione (antioxidant defence), a mechanism that competes for the precursor molecule homocysteine with SAM biogenesis metabolism (Ruiter *et al*., 2016; García‐Giménez *et al*., 2017) Arsenic requires SAM for its metabolization, leading to DNMTs having fewer methyl donors available (Zhao *et al*., 1997)	↓ DNA methylation
**As**	↑ Activity of 6‐mA demethylases	Up‐regulation of 6‐mA demethylases coding genes and increased stability of 6‐mA DNA demethylase enzymes (Cui *et al*., 2022)	↓ DNA methylation (adenines)
	↓ Activity of histone acetyltransferase and of other histone‐modifying enzymes	Direct inhibition of histone acetyltransferase hMOF (Liu *et al*., 2015a) Result of oxidative inactivation of enzymes (García‐Giménez *et al*., 2021) Arsenic competitively binds to zinc finger domains, leading to reduced enzyme activity and an increase in enzymatic degradation (Tam *et al*., 2017) Arsenic depuration depletes SAM pools leading to reduced histone methyltransferase ability to catalyse histone methylation (Kreuz & Fischle, 2016)	↓ H4 acetylation and other histone marks establishment
	↓↑ Histone writers/erasers enzymes activity	Arsenic compounds can directly interact with the enzymes responsible for histone modifications, either increasing and/or inhibiting their activity leading to altered histone modification patterns (Meakin *et al*., 2017; Valles *et al*., 2020)	↓↑ Histone modifications
		Arsenic interferes with expression of genes involved in epigenetic regulation (e.g. *SIRT1*) (Herbert *et al*., 2014), that in turn leads to altered histone‐modifying enzymes activity (Wang *et al*., 2019)	
**Cr**	↓↑ miRNA and long ncRNA expression	Cr caused differential expression of several miRNAs, along with changes in the expression profile of miRNAs and long ncRNAs (Chandra *et al*., 2015; Pratheeshkumar *et al*., 2016; Hu *et al*., 2019; Speer *et al*., 2022)	↓↑ miRNAs and long ncRNAs; ∆ expression of miRNA and long ncRNA classes

Owing to its high toxicity and recurrent prevalence in aquatic environments, cadmium (Cd) has been investigated for its ability to exert epigenetic toxicity through changes in DNA methylation patterns (Pan *et al*., 2010). For example, Cd‐exposed zebrafish embryos (183.3–5860 μg l^−1^) showed altered site‐specific DNA methylation that led to changes to cellular differentiation and programming during embryogenesis, ultimately compromising antioxidant physiology and neurobehavioural changes in adult stages (Ruiter *et al*., 2016). Exposed *Anguilla anguilla* (0.4–4 μg l^−1^) showed an increase in global DNA methylation and in the methylation level of transposable elements, accompanied by a decrease in total RNA content, arguably hampering their ability to respond to the Cd stress (Pierron *et al*., 2014). Chronically Cd‐exposed (10 mg kg^−1^ of soil) *Lumbricus terrestris* also showed a long‐lasting increase in total methylation levels (Šrut, Drechsel & Höckner, 2017). An inverse trend was observed in juvenile Nile tilapia (*Oreochromis niloticus*) exposed to Cd (10 and 50 μg l^−1^) as global DNA methylation levels decreased coupled with DNMTs and TET mRNA expression down‐ and up‐regulated, respectively (Hu *et al*., 2021). From this evidence, one can conclude that the effects of Cd in DNA methylation may be species, tissue, development and exposure specific.

Cd‐induced changes to the DNA methylation landscape of exposed organisms have been explained through several mechanisms. Acute Cd exposure inhibits DNMT activity (Takiguchi *et al*., 2003), while chronic Cd exposure seems to lead to stimulation of DNMTs‐coding genes and a consequent increase in DNA methylation. Changes in DNA demethylases can also be a response to Cd toxicity, with TET enzymes being directly inhibited through an inhibition mechanism not yet characterized but that potentially involves Cd interfering with the zinc finger domains of TET proteins (Xiong *et al*., 2017; Keith *et al*., 2021). The described inhibition of DNMTs and TET enzymes by Cd is not surprising since this metal frequently inhibits enzymes as part of its toxicity, owing both to its ability bind to motifs/domains critical to enzymatic activity (Filipič, 2012; Keith *et al*., 2021) and to the generation of oxidative stress leading to decreased enzymatic activity (Cuypers *et al*., 2010). Additionally, the DNMT methyl donor SAM can be depleted due to an increased need for reduced glutathione production following detoxifying conjugation with Cd or as an outcome of the antioxidant response (Ruiter *et al*., 2016). Reduced glutathione production requires the activation of the transsulfuration pathway that in turn competes with SAM production (*via* the methionine cycle) for the common precursor metabolite homocysteine (Kloypan *et al*., 2015; Ruiter *et al*., 2016; García‐Giménez *et al*., 2017). Cd‐driven oxidative stress interferes with DNMTs DNA‐binding capacity (Franco *et al*., 2008), favours the passive conversion of 5‐mC to 5‐hmC (Belzil *et al*., 2016), and compromises the ability of DNA to function as a substrate for DNA methylation machinery through inducing oxidative DNA damage, mutations and chromosomal alterations (Wachsman, 1997; Pogribny *et al*., 2005; Tunc & Tremellen, 2009). On the other hand, Cd‐induced oxidative DNA strand breaks can promote DNA methylation through the increased recruitment of DNMTs during the DNA repair process, thus reshaping the DNA methylation landscape and offering a reasonable explanation for Cd‐induced hypermethylation (Hayashi, Hishikawa & Itoh, 2019; Jiang *et al*., 2020).

Like Cd, arsenic (As) exposure leads to DNA hypomethylation though SAM depletion (Li *et al*., 2009; Hamdi *et al*., 2012; Chakraborty *et al*., 2022). However, this is not *via* competition with the reduced glutathione production process but rather because detoxification metabolism of this trace element requires SAM to methylate and then depurate As compounds (Zhao *et al*., 1997; Takiguchi *et al*., 2003; Abuawad *et al*., 2021). Therefore, the use of SAM for As detoxification may impose cofactor limitations on the activities of DNMTs and other cellular methyltransferases including histone methyltransferases (see below).

Changes in DNA methylation ‘eraser’ enzyme mRNA expression, stability and enzymatic activity can also influence DNA methylation levels. Arsenic increases the stability of 6‐mA DNA demethylase enzymes and consequently their activity (Cui *et al*., 2022), concerning 5‐mC, it directly inhibits the activity of TET enzymes through binding to their zinc finger domains, impairing their catalytic efficiency (Liu *et al*., 2015b; Wang, Wang & Zhang, 2021). Similarly to Cd, As exposure can lead to a variety of different epigenetic outcomes, with studies demonstrating that As exposure is associated with both hypo‐ and hypermethylation of the DNA, possibly in a tissue‐specific manner. Taken together, Cd and As compounds give valuable insights into the mechanisms behind DNA methylation responses to environmental contaminants. For example, a parallel could be drawn between the previously discussed mechanisms by which Cd and As modulate 5‐mC DNMTs and the ways in which DNMTs responsible for DNA 6‐mA can be affected by a multitude of contaminants (e.g. Wamucho *et al*., 2023; Yin *et al*., 2023).

Histone modifications can also occur due to environmental contaminants exposure, and As compounds are known modulators in this regard (Chakraborty *et al*., 2022). For instance, arsenic trioxide (2000 mg l^−1^) decreases global histone H4 acetylation at lysine 16 through inhibition of the activity of histone acetyltransferase hMOF in HeLa and HEK293T cells (Liu *et al*., 2015a). Sodium‐arsenite exposed zebrafish individuals (500 μg l^−1^) were found to have increased histone 3 lysine 4 trimethylation (Valles *et al*., 2020), often associated with up‐regulation of genes related to synaptic transmission and neuronal activity (Dincer *et al*., 2015). The reported changes to the histone modification landscape of exposed organisms are likely the result of an interaction between As and histone methyltransferases and demethylases, both regulators of histone methylation (Valles *et al*., 2020). In fact, As compounds were found to modulate/interact with a wide range of histone modifications such as histone methylation, acetylation, phosphorylation, and SUMOylation, often leading to altered chromatin structure, chromosomal alterations and altered gene expression (Bannister & Kouzarides, 2011; Burman *et al*., 2015). The exact mechanisms behind these links are not fully understood (Bhattacharjee & Paul, 2020), but some are known. The metabolism of As compounds interferes with the transcription of genes responsible for expression of histone modifier enzymes (Alamdar *et al*., 2017; Tu *et al*., 2018), and with the expression of several critical genes involved in epigenetic regulation (e.g. *SIRT1*), thus modulating chromatin structure through stimulation/repression of histone deacetylation or methylation (Herbert *et al*., 2014). Arsenic can also cause inactivation of histone modifier enzymes, *via* oxidation, to which the susceptibility of each of the multiple enzymes responsible for histone modifications differs (Zhou *et al*., 2008; Ramirez *et al*., 2008; Bhattacharjee & Paul, 2020; García‐Giménez *et al*., 2021). An additional mechanism is the competitive binding of As to their zinc finger domain motifs (Liu *et al*., 2015a; Tam *et al*., 2017). Depletion of the SAM pool within the cell also interferes with histone methylation, and offers an additional mechanism through which As can influence histone modifications (Kreuz & Fischle, 2016; García‐Giménez *et al*., 2021). Additionally, it seems that sex‐specific trends exist in humans and human‐related models (Chervona *et al*., 2012; Bhattacharjee & Paul, 2020), as well as in environmentally relevant models (Valles *et al*., 2020), regarding histone modifications elicited by As exposure, a factor that needs to be taken into consideration.

The mechanistic link between exposure to environmental contaminants and histone modifications is still poorly understood, especially in an ecotoxicological context. However, the current knowledge of As effects on histone modifications can serve as a valuable ‘roadmap’ for future studies focusing on histone modification modulation by other environmental contaminants, especially for studies using environmentally relevant models.

Concerning ncRNAs, it seems that miRNAs respond more readily to chemical exposures. Changes in miRNAs levels and in the classes of miRNAs expressed have been found in a variety of organisms and cell cultures in response to exposures to a multitude of environmental stressors (Saul *et al*., 2014; Fay *et al*., 2018; Lee *et al*., 2018; Zeng *et al*., 2019; Kwon *et al*., 2022).

An interesting example of ncRNAs modulation occurs in chromium (Cr)‐exposed organisms, where the profile of ncRNAs being expressed is altered upon exposure. In *D. melanogaster* exposed to Cr (5000–20000 μg l^−1^), several miRNAs showed differential expression that are involved in the regulation of genes responsible for DNA damage repair, detoxification, development and cell differentiation pathways (Chandra, Pandey & Chowdhuri, 2015). Similarly, several studies identified altered expression signatures of miRNAs and long ncRNAs in cell lines exposed to Cr (65–260 μg l^−1^), many of them targeting genes related to DNA damage, cell cycle regulation and antioxidative capacity (Pratheeshkumar *et al*., 2016; Hu *et al*., 2019; Speer *et al*., 2022; Zhang *et al*., 2023). In fact, fundamental research on ncRNAs in non‐human models is quite scarce and consequently the mechanisms behind ncRNAs modulation are still largely unknown. It is possible that environmental exposure to contaminants modulates ncRNAs expression patterns through interference with the other two epigenetic layers (see above), due to ncRNAs expression being under epigenetic control (Morris & Mattick, 2014).

## VI. FUTURE DIRECTIONS ON (ECO)TOXICOEPIGENETICS RESEARCH

This review demonstrates that before we can identify epigenetic biomarkers suitable for ecological monitoring and assessment, the generation of a robust body of fundamental knowledge is critically needed. We make the following recommendations for investment in future research:

(1) Narrowing the focus to a small array of ecologically relevant model species is perhaps the most sustainable approach, considering that few have been sequenced and even fewer have reasonably annotated genomes. Well‐established aquatic ecotoxicology model species with good‐quality genome assemblies, gene annotation and with known epigenome characteristics (e.g. *Danio rerio* and *Daphnia magna*) could serve as useful starting points for future research on the mechanisms behind epigenome modulation. Additionally, invasive species (e.g. *Procambarus virginalis*) could prove to be useful models to clarify the role of epigenetic mechanisms in gene regulation and adaptation to stress. However, additional fundamental research is required to obtain functionally annotated genomes and to understand epigenome dynamics considering sex, genetic variability, and tissue and developmental specificity. This knowledge will be a critical step towards feasibly appraising intricate mechanisms of ecotoxicity and the pathways behind differential toxicity sensitivity. Another important perspective that should be considered is that most studies focusing on epigenetic effects of environmental contaminants have used asexual models – an understandable choice in the analysis of epigenetic data since it eliminates any ‘noise’ from genetic variability. To integrate epigenetic‐based endpoints/biomarkers more fully into Environmental Risk Assessment (ERA), future studies should also focus on organisms that are representative of the environment and ecosystems under analysis, which often will reproduce sexually. A more diverse pool of rationally selected species will provide a wider view across different organisms (e.g. vertebrates, invertebrates, plants), to enable disentangling epigenetic phenomena in natural populations.
(2) It is difficult to ascertain which epigenetic changes are adaptive responses to contaminants exposure, i.e. changes that benefit organisms in dealing with toxicity, from those that effectively lead to adverse phenotypic changes (in other words an adverse outcome). This brings added challenges to the development of epigenetic‐based ecotoxicological biomarkers of exposure and effect. Thus, prioritization of studies that identify methylation in specific genes and/or genomic regions, as well as that acknowledge the complexity of interactions between contaminants and epigenetic machinery, is needed. We also require correlated evidence covering all three epigenetic layers, since the silencing or activation of a specific gene or genomic region may be the result of synergistic action of DNA methylation, histone modifications and/or ncRNAs. Additionally, the establishment of cause–effect relationships explaining how a biological system responds to epigenetic perturbations caused by environmental contaminants would aid the development of epigenetic‐based ecotoxicological biomarkers.
(3) We need to move beyond exploring differentially methylated nucleotides and other epigenetic marks in coding regions to investigating the regulatory outcomes of contaminant‐induced epigenetic changes in non‐coding regions of organisms, for example in transposable elements, as these elements are capable of having an impact on the expression of nearby genes (Lykoskoufis *et al*., 2024) and are thought to have important roles in stress response mechanisms (Horváth, Merenciano & González, 2017; Pappalardo *et al*., 2021). Epigenetic marks are essential for the regulation of repetitive sequences (e.g. transposable elements and satellite DNA), ncRNAs‐coding loci (e.g. long intergenic non‐coding RNAs and intergenic miRNAs), and other regulatory elements (e.g. enhancers, insulators, silencers) located in non‐coding DNA regions. Contaminant‐induced changes in the epigenetic landscape of these non‐coding regions could lead to increased genomic instability and chromosomal alterations, which necessarily impacts gene expression and normal cellular function.
(4) Controlling the diversity of exposure methodologies towards building solid information on the epigenetic responses to a given contaminant class in each model organism will be crucial to build a basis for feasible future predictions that are a cornerstone of prospective (regulatory) ERA. Indeed, distinct exposure designs, e.g. exposure length, contaminant levels, developmental stage, genetic variability, and test incubation conditions, cannot be dissociated from the consequent epigenetic effects. For example, longer exposure times to a contaminant can lead to the opposite epigenetic outcome than a shorter exposure window. The use of standard test conditions for model species and the limiting of extrapolations among species or among organisms of the same species tested under different conditions should be the gold standard for (eco)toxicoepigenetics.
(5) Avoiding research focusing solely on epigenetic responses by extending our understanding of their consequences, namely concerning gene expression and phenotypes, is of major importance to render the incorporation of epigenetic biomarkers into ERA a reachable reality. Here frameworks like the AOP framework proposed by Ankley *et al*. (2010) and the framework put forward by Bowers & McCullough (2017) can assist in designing future (eco)toxicoepigenetics studies. Briefly, the AOP framework and to some extent that of Bowers & McCullough (2017) allow for an analytical construct that establishes causal links between molecular initiating events, key events spanning various levels of biological organization, and the final ecologically relevant adverse outcome. The use of such frameworks will undoubtedly aid in the integration of molecular responses with downstream phenotypic consequences, from the gene expression level to higher levels of biological organization and ecologically relevant outcomes (e.g. fertility and/or fecundity, population growth, behaviour).

## VII. CONCLUSIONS

(1) The plethora of epigenetic alterations that arise in response to environmental stimuli, such as exposure to environmental contaminants, is an interesting avenue of research for (eco)toxicology studies. Epigenetic data have tremendous implications for this scientific field, and for its interconnections with regulatory ERA. In particular, the prospect of using epigenetic‐based biomarkers has been a focal point of (eco)toxicoepigenetics research, leading to the identification of novel putative links between environmental exposures, disease susceptibility and public/environmental health.
(2) It is critical to acknowledge the gap between what is known for human health and the much lower understanding of the mechanistic basis of epigenetic responses to stress in ecologically relevant models. At present, very little is known on the modulation of the epigenetic machinery of eco(toxico)logical model species by environmental contaminants, concerning epigenetic mechanisms individually and more so their crosstalk regulating gene expression responses to stress.
(3) Herein, we reviewed current knowledge on epigenetic regulation and on the mechanisms behind their modulation by environmental contaminants. This review will hopefully stimulate much needed fundamental research focusing on the mechanistic links between exposure to environmental contaminants and epigenetic outcomes on ecotoxicology models.
(4) Future studies should focus on the characterization of the genomes and epigenomes of ecotoxicological models as a basis for a better understanding of the mechanisms by which environmental contaminants modulate epigenetic marks and related machinery, while reinforcing experimental standardization efforts. Additionally, linking epigenetic responses to downstream phenotypic effects will be paramount to making the incorporation of epigenetic biomarkers into ERA a reality.

## Supporting information


**Table S1.** Main epigenetic effects of environmental contaminants identified as priority hazardous substances by the EU Water Framework Directive on *in vivo* animal models relevant for ecotoxicology.
