# Supplementary material for: Integration of epigenetics into ecotoxicology: insights and fundamental research needs
Source: Biol Rev Camb Philos Soc. 2025 Nov 16;101(2):826–47. doi: 10.1111/brv.70105 (PMC12965860; doi:10.1111/brv.70105)
Supplement: Supplementary file 1 — Table S1. Main epigenetic effects of environmental contaminants identified as priority hazardous substances by the EU Water Framework Directive on in vivo animal models relevant for ecotoxicology. [file BRV-101-826-s001.docx]

Table S1. Main epigenetic effects of environmental contaminants identified as priority hazardous substances by the EU Water Framework Directive on *in vivo* animal models relevant for ecotoxicology. Most of the available work on epigenetic effects of these environmental contaminants focuses on human-related models and/or on *in vitro*-cultured cell models. Epigenetic effects of environmental contaminants are given for DNA methylation, histone modifications and non-coding RNAs. When applicable, putative mechanisms underlying epigenetic changes are listed in parentheses; NA = lack of relevant information. DNMT, DNA methyltransferase; miRNA, microRNA; SAM, S-adenosylmethionine; TET, ten-eleven translocation methylcytosine dioxygenase.

| **Name of priority hazardous substance** | **DNA methylation** | **Histone modifications** | **Noncoding RNAs** | **Experimental models used** | **References** |
| --- | --- | --- | --- | --- | --- |
| Anthracene and other polycyclic aromatic hydrocarbons (PAHs) | Global and locus-specific DNA methylation modulation (increased DNMT1 and glycine N-methyltransferase expression) | Increase in HDAC1 expression | Overexpression of miR-203 (upregulation of genes *DICER1* and *Ago2*, involved in miRNA biogenesis) | *Danio rerio*;  *Caretta caretta*;  *Mus muluscus* | Mirbahai *et al.* (2011); Tiwari & Gupta (2014); Cocci *et al.* (2018) |
| Brominated diphenylethers | Locus-specific DNA methylation modulation (decreased DNMT3ba expression) | Altered histone acetylation and methylation levels | Altered miRNAs expression profiles | *Danio rerio*;  *Ctenopharyngodon idell* | Gan *et al.* (2016); Zhao *et al.* (2017); Blanc *et al.* (2021); Sun *et al.* (2023*b*) |
| Cadmium and its compounds | Global and locus-specific DNA methylation modulation (cadmium-induced changes on DNA methylation levels is dependent on the time of exposure and DNMT activity inhibition) | Increased H3K4me3 histone modifications coupled with decreased H3K9me3 and H3K27me3 marks  and reduced histone acetylation (altered expression of histone methylation-related genes) | Altered miRNAs and long non-coding RNAs expression profiles | *Daphnia pulex*;  *Danio rerio*;  *Tegillarca granosa*;  *Chironomus riparius*; *Mus muluscus*; *Drosophila melanogaster* | Martínez-Guitarte *et al.* (2012); Bao *et al.* (2014); Chen *et al.* (2015); Guo *et al.* (2018); Sun *et al.* (2023*a*) |
| Chloroalkanes | NA | NA | NA | NA | NA |
| Di(2-ethylhexyl)phthalate (DEHP) | Global and locus-specific DNA methylation modulation | Reduction in histone demethylase expression | Reduced expression of MiR-146a and other miRNAs with altered expression patterns | *C. elegans*;  *Danio rerio*;  *Mus muluscus* | Li *et al.* (2018); Guo *et al.* (2020); Du *et al.* (2021); Liu *et al.* (2021) |
| Endosulfan | NA | NA | NA | NA | NA |
| Hexachlorobenzene | NA | NA | NA | NA | NA |
| Hexachlorobutadiene | NA | NA | NA | NA | NA |
| Hexachlorocyclohexane | NA | NA | NA | NA | NA |
| Mercury and its compounds | Global and locus-specific DNA methylation modulation (DNMT activity modulation) | Increased histone H3K27 trimethylation, and decreased histone H3 acetylation (altered expression of histone-modifying enzymes) | Altered miRNA and long noncoding RNAs expression profiles | *Lumbricus terrestris*; *Mus muluscus*; *C. elegans*; *Danio rerio*; *Neovison vison* | Onishchenko *et al.* (2008); Wrobel *et al.* (2011); Basu *et al.* (2013); Rudgalvyte *et al.* (2013); Carvan *et al.* (2017); Cao *et al.* (2019); Go *et al.* (2021) |
| Nonylphenols | NA | NA | NA | NA | NA |
| Pentachlorobenzene | NA | NA | NA | NA | NA |
| Tributyltin compounds | Global DNA hypomethylation (depletion of intracellular SAM, leading to a lack of methyl donors, DNMT1 activity inhibition) | NA | Altered miRNAs, long noncoding RNAs, and circRNAs expression profiles | *Sebastiscus marmoratus*;  *Tritia mutabilis*; *Danio rerio* | Wang *et al.* (2009); Bouwmeester *et al.* (2016); Cocci *et al.* (2021); Li *et al.* (2024) |
| Trifluralin | NA | NA | NA | NA | NA |
| Dicofol | NA | NA | NA | NA | NA |
| Perfluorooctane sulfonic acid (PFOS) and its derivatives | Global and locus-specific DNA methylation modulation (decreased DNMT3a expression coupled with increased TET1 expression) | Altered histone acetylation levels (altered histone-modifying enzymes activity, due to oxidative stress and/or reduced gene expression) | Altered miRNA expression profiles | *Mus muluscus*; *Rattus norvegicus*; *Danio rerio*; *Dugesia japonica* | Zhang *et al.* (2011) Zhang *et al.* (2019); Wang *et al.* (2015); Blanc *et al.* (2017, 2019); Li *et al.* (2019); Wen *et al.* (2020) |
| Quinoxyfen | NA | NA | NA | NA | NA |
| Dioxins and dioxin-like compounds | Locus-specific DNA methylation modulation (increased DNMT3A expression) | Altered histone acetylation levels (altered histone-modifying enzymes activity) | Reduced expression of the long noncoding RNA H19 | *Danio rerio*; *Mus muluscus* | Olsvik *et al.* (2014); Gao *et al.* (2016); Yuan *et al.* (2016); Zhang *et al.* (2018) |
| Hexabromocyclododecanes (HBCDDs) | Locus-specific 6-mA methylation modulation | NA | Reduced expression of miRNA-1 | *Danio rerio*; *Rattus norvegicus* | Wu *et al.* (2016*a*); Holuka *et al.* (2023) |
| Heptachlor and heptachlorepoxide | NA | NA | NA | NA | NA |
